# Molecular Characterization, Expression Pattern and Function Analysis of Glycine-Rich Protein Genes Under Stresses in Chinese Cabbage (*Brassica rapa* L. ssp. *pekinensis*)

**DOI:** 10.3389/fgene.2020.00774

**Published:** 2020-07-23

**Authors:** Xiaonan Lu, Yaxiong Cheng, Ming Gao, Meilan Li, Xiaoyong Xu

**Affiliations:** College of Horticulture, Shanxi Agricultural University; and Collaborative Innovation Center for Improving Quality and Increasing Profits of Protected Vegetables in Shanxi, Taigu, China

**Keywords:** Chinese cabbage, *GRP* gene family, expression analysis, biotic and abiotic stress, overexpression, *RBP-GRP*, resistance

## Abstract

Plant Glycine-rich proteins (GRP), a superfamily with a glycine-rich domain, play an important role in various stresses such as high or low temperature stress and drought stress. *GRP* genes have been studied in many plants, but seldom in Chinese cabbage (*Brassica rapa* L. ssp. *pekinensis*). In this study, a total of 64 GRP genes were identified in Chinese cabbage by homology comparative analysis. The physical and chemical characteristics predicted by ProtParam tool revealed that 62.5% of BrGRPs were alkaline, 53.1% were stable, and 79.7% were hydrophilic. Conserved domain analysis by MEME and TBtools showed that 64 BrGRPs contained 20 of the same conserved motifs, based on which BrGRPs were classified into five main classes and four subclasses in class IV to clarify their evolutionary relationship. Our results demonstrated that The *BrGRP* genes were located on ten chromosomes and in three different subgenomes of Chinese cabbage, and 43 pairs of orthologous *GRP* genes were found between Chinese cabbage and *Arabidopsis*. According to the transcriptome data, 64 *BrGRP* genes showed abnormal expression under high temperature stress, 52 under low temperature stress, 39 under drought stress, and 23 responses to soft rot. A large number of stress-related *cis-*acting elements, such as DRE, MYC, MYB, and ABRE were found in their promoter regions by PlantCare, which corresponded with differential expressions. Two *BrGRP* genes-*w546* (*Bra030284*) and *w1409* (*Bra014000*), both belonging to the subfamily Subclass IVa *RBP-GRP* (RNA binding protein-glycine rich protein), were up-regulated under 150 mmol⋅L^–1^ NaCl stress in Chinese cabbage. However, the overexpressed *w546* gene could significantly inhibit seed germination, while *w1409* significantly accelerated seed germination under 100 mmol⋅L^–1^ NaCl or 300 mmol⋅L^−1^ mannitol stresses. In short, most *BrGRP* genes showed abnormal expression under adversity stress, and some were involved in multiple stress responses, suggesting a potential capacity to resist multiple biotic and abiotic stresses, which is worthy of further study. Our study provides a systematic investigation of the molecular characteristics and expression patterns of *BrGRP* genes and promotes for further work on improving stress resistance of Chinese cabbage.

## Introduction

Plant glycine-rich protein (GRP) is a class of proteins consisting of glycine-rich repeat sequences. The first glycine-rich cell wall protein, PtGRP1, was isolated from *Petunia hybrida* in 1986 (Condit and Meagher, 1986; Chen et al., 2020) and similar proteins have since been found in almost all plants, such as *Zea mays*, *Oryza sativa*, *Arabidopsis thaliana*, and *Nicotiana tabacum* (Mangeon et al., 2009).

Plant GRPs can be divided into five main classes based on their primary structure, the conserved domain, and the arrangement of glycine repeats (Fusaro et al., 2001; Bocca et al., 2005). The class I family contains a typical structural feature of the GRP protein in which the glycine-rich repeats mostly appear as (Gly)n-X, where n is generally an odd number and X can be any amino acid. At the same time, the N-terminus contains or does not contain a signal peptide sequence. For example, PtGRP1 has 67% of glycine in glycine repeat (Gly)n-X (Mangeon et al., 2009). Class II of GRPs are similar to class I at the N-terminus, and contains a cysteine-rich polypeptide chain at the end of the C-terminal glycine repeat region that may play an important role in the pathogen-related process, such as *AtGRP3* (Park et al., 2001). The glycine content of class III is lower than that of the first two classes, but the oleosin domain is the unique motif for this class. For example, oleosin-GRPs have an oleosin conserved area and are located in an oil-rich cell structure of tapetum, which mainly plays a role in stabilizing the triglyceride and phospholipid bilayer in the membrane structure (Ferreira et al., 1997; Murphy et al., 2001). Class IV GRPs, also named RNA-binding proteins (RBPs), have no signal peptide at their N-terminus. These proteins have other structures besides the glycine-rich sequences, such as the RNA-recognition motif (RRM), cold-shock domain (CSD), and CCHC (CCHC = C-X_2_-C-X_4_-H-X_4_-C; C = Cys, H = His, X = variable amino acid) zinc finger structure. Class IV can be divided into four subgroups based on its domains: Subclass IVa (single RRM), Subclass IVb (single RRM and CCHC zinc-finger motif), Subclass IVc (cold shock domain and two or even more zinc-fingers), and Subclass IVd (two RRM motifs) (Mangeon et al., 2010). These RNA recognition domains can usually recognize each other or interact with proteins without any RNA recognition domain to bind to their target (Dreyfuss et al., 2002; Lorkovic and Barta, 2002). Class V GRPs are similar to the members of class III as the glycine repeats are arranged differently and presented with the mixed mode of (GGX)n and (GXGX)n (Bocca et al., 2005).

Plant *GRP* genes are often specifically expressed and play different roles in different development stages and tissues (Czolpinska and Rurek, 2018). The GRP proteins of classes I and II are active components of cell walls, which play a positive regulatory role in plant cell division and organ differentiation (Czolpinska and Rurek, 2018). The AtGRPs of class II can interact with the cell wall-associated receptor protein kinase AtWak1 to participate in the signal transduction to prevent viruses from invading plants (Park et al., 2001). The GRPs of class III regulate pollen development and hydration, while class IV GRPs with an RNA recognition domain and are involved in molecular processes such as alternative splicing or transcriptional regulation; the latter also play an important role in stomatal regulation and seed and stamen development (Winter et al., 2007; Czolpinska and Rurek, 2018). An oleosin-*GRP BrGRP17* gene from Chinese cabbage showed higher expression levels in the flower buds of male fertile plants than in sterile ones (Xu et al., 2013). The deletion mutant of RNA-binding glycine-rich protein five (*Atrbg5*) also yielded shorter roots, smaller leaves, and shorter flower axes, but overexpressed *AtRBG5* promoted cell elongation and tissue growth in *Arabidopsis* (Mangeon et al., 2009). In summary, the function of the plant *GRP* gene family varies across plant growth and development.

Besides that, plant GRPs are also involved in responses to various abiotic stress such as salt, drought, and temperature, and may play an important role in resisting adversity stress. A notable increase of *RB-GRP* (*RNA-Binding GRP*) was observed in *S. Bicolor* seedlings subjected to NaCl treatments with 1 M and 500 mM (Aneeta et al., 2002). Overexpressing *AtRZ-1a*, a zinc finger-containing GRP, lead to retarded germination and seedling growth under salt or dehydration stress conditions in transgenic *Arabidopsis* (Kim et al., 2005) while the loss-of-function mutants of *AtRZ-1a* germinated earlier and grew faster than the wild-type plants under the same conditions (Kim Y.O. et al., 2007). *NtGRP-1a* was up-regulated under drought stress and could be maintained for 3 to 6 days (Chen et al., 2010). The ryegrass *LPGRP1* gene was up-regulated under cold stress treatment (Shinozuka et al., 2006). The expression level of the *AtRBG7* gene was higher at low temperatures than high ones (32°C) (Wienkoop et al., 2008). Overexpression of the *AtRBG7* gene increased cold tolerance but inhibited seed germination and plant growth under drought stress (Kim et al., 2008), while overexpression of *AtRBG2* also increased cold tolerance and lead to a higher germination rate under salt stress in *Arabidopsis* (Kim J.Y. et al., 2007). Ectopic expression of *AtRBG2* and *AtRBG7* in rice could also increase crop yield under drought stress (Yang et al., 2014). In other words, different *GRP* genes may play different functions in plant responses to various stresses, and expression dynamics may vary under the same stress.

Up to date, the *GRP* gene family has been studied in many plants (Kar et al., 2012; Zhang et al., 2014; Krishnamurthy et al., 2015; Lu et al., 2019), however, the *BrGRP* gene family has not been reported on Chinese cabbage. In this study, 64 *GRP* genes in Chinese cabbage were identified based on the existing BRAD genome database^1^ (Cheng et al., 2011) and their phyletic evolution, module prediction, and chromosomal localization were further investigated. Moreover, the expression patterns of these *BrGRP* genes were also detected in different tissues and various abiotic stresses based on open transcriptome databases, their functions, and evolutions in development and stress response were also discussed. Furthermore, the function of two *BrGRP* genes screened from the normalized cDNA library of male sterile bud from Chinese cabbage flower (Li et al., 2018) were further characterized under salt treatment. These results provide valuable information for further exploration into the function of *BrGRP* genes in Chinese cabbage.

## Materials and Methods

### Identification and Sequence Conservation of *GRP* Genes in Chinese Cabbage

The *GRP* genes in *Arabidopsis thaliana* (TAIR database^2^) (Lamesch et al., 2012) were employed as a query to search against Chinese cabbage genome database (BRAD v1.5^1^) (Cheng et al., 2011). A total of 64 genes in Chinese cabbage genome were identified as possible members of *BrGRP* genefamily using the Blastn program. All putative protein sequences of *GRP* genes in *Arabidopsis thaliana* and Chinese cabbage were identified for the GRP conserved domain using the NCBI Conserved Domain Database^3^, and Pfam^4^. The sequences without glycine-rich protein domains were removed. Finally, the nucleotide and deduced amino acid sequences of *BrGRP* genes were confirmed for further analysis.

### Characterization Analysis and Subcellular Localization Prediction of BrGRPs

Physical and chemical characteristics of the BrGRP sequences – i.e., the molecular weight (MW), theoretical point (pI), instability index, aliphatic index, and grand average of hydropathicity (GRAVY) – were further analyzed using the ProtParam tool in ExPASy^5^. The subcellular localization of these BrGRPs was predicted by the ProtComp tool on Softberry^6^.

#### Multiple Sequence Alignment and Phylogenetic Analysis

Multiple sequence alignments of the published protein sequences were performed by Clustal X with default parameters, including 13 OsGRPs (Krishnamurthy et al., 2015), 18 MaGRPs (Zhang et al., 2014; Krishnamurthy et al., 2015), 32 GrGRPs (Yang et al., 2019), 37 GaGRPs (Yang et al., 2019) and 9 ItGRPs (Lu et al., 2019) from rice^7^, maize^8^, *Gossypium raimondii*^9^, *Gossypium arboreum*^9^, and sweet potato^10^, respectively. The phylogenetic tree was constructed by the Neighbor-Joining method (NJ) and Maximum likelihood (ML) on MEGA X (Kumar et al., 2018) and the check parameter bootstrap value was set to 1000 times.

#### Analysis of the Conserved Domain and Gene Structure of *BrGRP* Genes

The structures of the coding/non-coding region of *BrGRP* genes were mapped by the software TBtools (Chen et al., 2020). Next, the conservative motifs were analyzed by MEME (version 5.0.3^11^), with the number of motifs set to 20 and the other parameters set to default values. The LOGO of conservative motifs was listed, and TBtools was used to export the corresponding Scalable Vector Graphics (SVGs) (Chen et al., 2020).

#### Identification of the Orthologous *BrGRP* Genes and Syntenic Analysis in Chinese Cabbage

According to the genomic and chromosome database (v2.5) of Chinese cabbage (Cheng et al., 2011, 2013) the identified *BrGRP* genes were located on ten chromosomes in three fractionated subgenomes, and the locations of the *BrGRP* genes on chromosome were visualized using MapChart (Voorrips, 2002). Syntenic relationships between BrGRP homologs of Chinese cabbage and *Arabidopsis thaliana* was defined in BRAD database (Cheng et al., 2011) and the corresponding circos were drawn out on TBtools (Chen et al., 2020).

### Expression Pattern of *BrGRP* Genes

To analyze the expression pattern of *BrGRP* genes in Chinese cabbage, transcriptome data from *B. rapa* “Chiifu” (Tong et al., 2013) and the inbred line “Fushanbaotou,” a typical heading Chinese cabbage (Wang et al., 2012) was used for gene expression profiling in eight tissues: callus, root, stem, leaf, flower, silique, rosette, and folding leaves.

The differences in *BrGRP* genes expression under biotic and abiotic stress were also analyzed based on the transcriptome data of Chinese cabbage under high temperature at 45°C (Dong et al., 2015), low temperature at 4°C (Zhang et al., 2016), drought (Guo et al., 2017), and soft rot stress (Liu et al., 2019). The expression levels of *BrGRP* genes were calculated with Fragments Per kb per Million read (FPKM) values (Mortazavi et al., 2008), and analyzed by STEM (Short Time-series Expression Miner) (Ernst and Bar-Joseph, 2006). Venn diagram and heat map were generated by TBtools (Chen et al., 2020) according to the differentially expressed data.

### Analysis of *Cis*-Acting Elements on *BrGRPs* Promoter

To further identify the *cis-*acting elements on the promoter regions of *BrGRP* genes, a 2-kb fragment upstream of the start codon was extracted by TBtools (Chen et al., 2020) and further identified by PlantCare (Lescot et al., 2002)^12^ with the adversity related *cis-*acting elements MYC (CANNTG), MYB (C/TAACNA/G), ABRE (ABA-responsive element, ACGT), LTR (low-temperature-responsive element, CCG AAA), DRE (CCGAC), W box (TTGACC), and TC rich repeats (GTTTTCTTAC). A Venn diagram was constructed using TBtools (Chen et al., 2020) based on the types of *cis-*acting element.

### Plant Material and qRT-PCR

The plump seeds of heading Chinese cabbage 18c901 (a homozygous inbred line) were planted in a tray filled with substrate and maintained in an intelligent chamber at 25°C, 2000 l× of light intensity, 16/8 h of light-dark period of, and 70% humidity. Plants with flower buds were treated with 150 mmol⋅L^–1^ NaCl solution. After 7 days’ treatment, the leaves, the whole flowers and roots were collected and quickly frozen by liquid nitrogen for quantitative PCR analysis.

Total RNA was extracted using Total RNA Kit (TIANGEN, China). All RNA was analyzed by agarose gel electrophoresis and then quantified with a Nanodrop ND-1000 spectrophotometer. DNA-free RNA was used to synthesize the first strand of cDNA using PrimeScript^TM^ RT Master Mix (Perfect Real Time) (Takara, Japan). The quantitative RT-PCR was run on ABI 7500 system using SYBR Green PreMix (Takara, Japan). The Chinese cabbage GAPDH (*AF536826.1*) was used as an internal control. The reactions were carried out by the following program: 94°C for 5 min; followed by 40 cycles of 94°C for 30 s, 55°C for 30 s, 72°C for 30 s; and 72°C for 5 min. Each reaction was performed in biological triplicates, and the data from real-time PCR amplification were analyzed using the 2^–ΔΔCT^ method. The primers sequences were shown in Supplementary Table S1.

### Generation of the Transgenic *Arabidopsis* Plants

The overexpression vector PHZM27-*w546*/*w1409* was constructed by inserting *w546*/*w1409* under the CaMV35s promoter and NOS terminator of PHZM27, and then transferred into the *Agrobacterium tumefaciens* strain GV3101 by electroporation. The flower-dip method was applied to transform *Arabidopsis* (Columbia-0, WT) according to the protocol described by Clough and Bent (1998). Transgenic *Arabidopsis* plants were determined on MS medium with by kanamycin screening assay (Park et al., 1998) and PCR analysis with 35S forward and *w546*/*w1409*-specific reverse primers (Supplementary Table S1).

### NaCl and Mannitol Stress in Transgenic *Arabidopsis* Plants

The homozygous seeds of T_3_ generation transgenic *Arabidopsis* were inoculated on 1/2 MS medium with 100 mmol⋅L^−1^ NaCl and 300 mmol⋅L^−1^ mannitol, respectively. The number of germination individuals were calculated every day. Each treatment contained 30 individuals and repeated three times. The cultivation environment was set as follows: temperature 22°C, light intensity 2000 l×, light-dark cycle 16/8 h, and humidity about 70%.

## Results

### Identification and Characterization of *BrGRP* Genes in Chinese Cabbage

To identify the *BrGRP* genes in Chinese cabbage, we first screened the *GRP* genes of *Arabidopsis* in TAIR and NCBI databases. A total of 41 *AtGRP* genes were obtained, of which 10 had no orthologous genes; a total of 64 *BrGRP* genes in BRAD database were identified from the remaining 31 *AtGRP* genes (Supplementary Table S2). Further prediction analysis revealed that the protein characteristics of these *BrGRP* genes showed great differences in molecular weight, theoretical point, etc. The length of amino acids ranged from 52 (Bra027145) to 2038 aa (Bra028693), and the theoretical point ranged from 4.59 (Bra008020) to 11.56 (Bra028691), with 62.5% BrGRPs with a theoretical point over 7. More detailed information including instability index, aliphatic index, and grand average of hydropathicity were also predicted. The results demonstrated that 46.9% of proteins showed an aliphatic index over 40, while 79.7% proteins showed a grand average of hydropathicity with a negative value. All these results suggested that these BrGRPs mainly existed on the stable alkaline hydrophilic protein.

Subcellular localization showed that most BrGRPs (36 of 65) were secreted into the extracellular matrix; 18 BrGRPs were localized in nuclear region, six in the mitochondria, two BrGRPs including Bra037177 and Bra031809 in the cytoplasm, and Bra031159 and Bra005945 in plasma membrane and chloroplast, respectively (Supplementary Table S2).

### Sequences Analysis of BrGRP Genes and Phylogenetic Relationship

**FIGURE 1:**
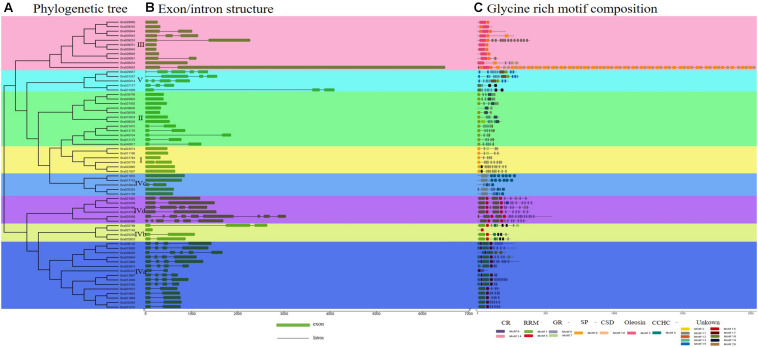
Phylogenetic relationship, gene structure, and conserved structural composition of 64 *BrGRP* genes in Chinese cabbage. **(A)** Phylogenetic tree created using Clustal X to align the amino acid sequences and MEGA X to generate the phylogenetic tree by a contiguous method with a calibration parameter of 1000. **(B)** Sequence structure distribution of *BrGRP* genes; the green and black boxes represent the exon and intron, respectively. Scale indicates 1.0 kb. **(C)** Schematic diagram of the conserved *GRP* protein motif in Chinese cabbage predicted by MEME. Colored boxes indicate different motifs; CSD, the cold-shock domain; RRM, RNA-recognition motif; oleosin, oleosin-conserved domain; CR, cysteine-rich domain; CCHC, zinc-finger; GR, glycine-rich domain. Scale bars represent 500 aa.

To gain insight into conserved domain of these BrGRPs, a total of 64 BrGRP genes showed similar conserved domains and 20 motifs were identified using MEME tool, including two glycine-rich motifs (motif 6, 7), two cysteine-rich domains (motif 9, 14), two RNA recognition motifs (motif 1, 5), one CCHC-Zinc finger structural motifs (motif 4), one cold shock motif (CSD) (motif 10), one oleosin lipid motif (motif 3), and one signal peptide motif (motif 8) (Figure 1C and Supplementary Figure S1). According to motif types, 64 BrGRPs were constructed a phylogenetic tree using the NJ method (Figure 1A) and were divided into five classes (Figure 1C). Class I contained six members, the N-termini with a typical glycine repeat structure followed by a signal peptide sequence or no signal peptide sequence. Class II was composed of 12 members, which comprised a cysteine-rich polypeptide chain at the end of C-terminal with a repeated glycine. Eleven BrGRPs were classed into Class III, whose glycine content was lower than that of the first two classes, and contained an oleosin domain. Class V (five members) had the lowest glycine content, and contained repeats of the pattern GGX/GX (X represents any amino acid), which coincided with class V of *Arabidopsis* demonstrated by Gilberto Sachetto (personal communicated).

In addition to the glycine-rich region, class IV (30 members) also had other structures, including one RNA recognition domain, cold shock domain, and CCHC-zinc finger domain. Based on these different conserved domains, class IV can be further divided into four subgroups (Subclass IVa, IVb, IVc, and IVd). Subclass IVa (15 members) contained a single RRM motif, while Subclass IVb (four members) included a single RRM and CCHC zinc-finger motif. Subclass IVc (five members) contained a cold shock domain and two or more zinc-fingers. Subclass IVd (six members) contained two RRM motifs. These five subclasses in *BrGRPs* were highly similar to those of previous studies in *Arabidopsis*, Rice and Maize (Mangeon et al., 2010; Krishnamurthy et al., 2015; Czolpinska and Rurek, 2018). The genes in the same subgroup shared a close phylogenetic relationship, high sequence similarity, and similar gene structures, revealing evolutionary conservation in the *GRP* gene family.

The distribution of the exon-intron structure in the 64 *GRP* genomic sequences was exhibited as Figure 1B. Twenty-three *BrGRP* genes had no introns, while the remained 41 *BrGRP* genes contained two or more exons, of which *Bra025568* contained nine exons, the most. For exon numbers, class I and II of *BrGRPs* had the least exons (one or two), classes III and V had 1–3 and 3–4 exons, respectively, while class IV appeared variety ranging from1 to 9 exons.

The phylogenetic relationships of 64 BrGRPs, 41 AtGRPsand other reported GRPs from cotton, rice, maize, and sweet potato were evaluated using NJ and ML method (Figure 1A and Supplementary Figure S2). The topological structure of the GRP gene phylogenetic tree constructed by the NJ method (Supplementary Figure S2A) and the ML method (Supplementary Figure S2B) is basically the same, and both have a high degree of confidence. The results showed that these BrGRPs were also classified into five classes (class I-V). Most GRPs belonged to class IV (30 BrGRPs, 18 AtGRPs 13 OsGRPs, 18 MaGRPs, 32 GrGRPs, 37 GaGRPs, and 9 ItGRPs), followed by class II (12 BrGRPs and 7 AtGRPs) and class III (11 BrGRPs and 7 AtGRPs), with several genes in class I (6 BrGRPs and 4 AtGRPs) and class V (5 BrGRPs and 5 AtGRPs). Interest, the reported GRPs from other species were all separated into the class IV which may suggest their most important function among five classes.

### Chromosome Localization and Orthologous Gene Analysis of BrGRPs in Chinese Cabbage

**FIGURE 2:**
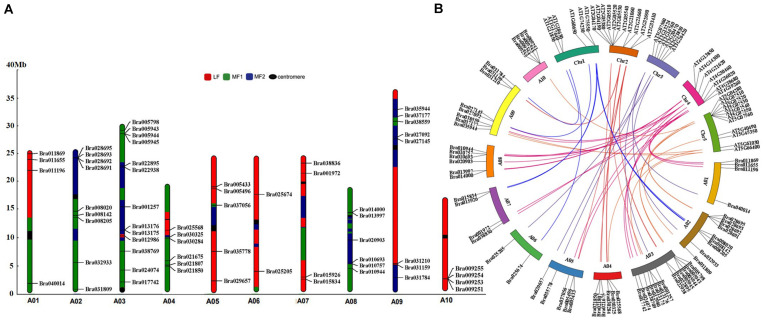
Chromosome distribution and syntenic analysis of GRP genes in Chinese cabbage. **(A)** MF1, the least fractionated subgenome; MF2, the medium fractionated subgenome; LF, the most fractionated subgenome; centromere, the position of the chromosome centromere. Syntenic analysis of GRP genes in Chinese cabbage and *Arabidopsis thaliana*. **(B)** The orthologous and paralogous GRP genes are localized on the chromosomes of Chinese cabbage (A01–A10) and *Arabidopsis thaliana* (Chr1–Chr5).

To examine their chromosomal distributions, the 64 *BrGRP* genes were mapped onto the chromosomes and three fractionated subgenomes of Chinese cabbage based on the *B. rapa* genome database (chromosome v1.5). Three *BrGRP* genes (*Bra040764*, *Bra040817*, *Bra039380*) could not be assigned to any chromosome, but the other 61 genes were successfully identified in 10 chromosomes; 13 *BrGRP* genes on Chromosome A03, while only one on Chromosome A06. Furthermore, 23 *BrGRP* genes were anchored on the least fractionated (MF1) subgenome, 18 genes on the medium fractionated (MF2) subgenome, and 20 genes on the most fractionated (LF) subgenome (Figure 2A and Supplementary Table S3).

Moreover, 43 pairs of *BrGRP* gene syntenic paralogs were found on different subgenomes of Chinese cabbage (Figure 2A and Supplementary Tables S3,S4). For example, *Bra022895* and *Bra021807* with the highest sequence similarities to *AT2G32690*, both located in the MF2 and MF1 subgenome, respectively. To further understand the duplication of the *BrGRP* genes during the whole genome duplication in Chinese cabbage, the orthologous analysis of GRP homologous genes was also compared between Chinese cabbage and *Arabidopsis thaliana* (Figure 2B and Supplementary Table S4). A total of 31 *AtGRP* genes were found to be orthologous in Chinese cabbage, and most of *GRP* genes in *Arabidopsis* had 1–6 orthologous genes in Chinese cabbage, but 10 *AtGRP* genes had no orthologous genes in Chinese cabbage. The results indicated that *BrGRP* genes had evolved during whole-genome duplication in Chinese cabbage, providing a valuable reference for uncovering the functions of *BrGRP* genes in Chinese cabbage.

### Expression Profiling of *BrGRPs* in Chinese Cabbage

Based on the published transcriptomic data, the expression of *BrGRP* genes was further analyzed in Chinese cabbage in different tissues and during two development stages under various abiotic and biotic stresses, and a set of *BrGRP* genes was identified to be abnormally expressed.

#### Different Tissues

**FIGURE 3:**
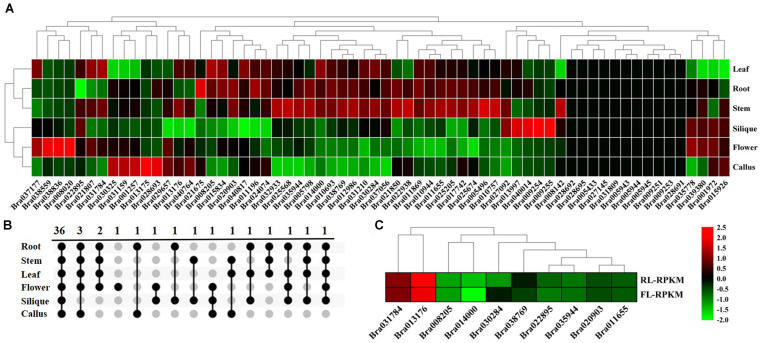
Expression analysis of *BrGRP* gene expression in Chinese cabbage. **(A)** Differential expression pattern of the *BrGRP* gene in different tissues of Chinese cabbage. **(B)** Venn diagram of *BrGRP* expression in six tissues of Chinese cabbage. Numbers represent the number of genes. **(C)** Differential expression pattern of the *BrGRP* gene of Chinese cabbage in the rosette and folding leaves.

The tissue-specific expression profiling of 64 *BrGRP* genes were detected in different tissues based on the transcriptome data from the Chinese cabbage line “Chiifu” (Tong et al., 2013; Figure 3A and Supplementary Table S5). *BrGRP* gene expressions were different among the root, stem, leaf, flower, silique and callus of Chinese cabbage, but 11 genes were found no expression in any tissue. Among them, *Bra031210* had the highest expression in four tissues, including the root, leaf, stem, and silique, while *Bra038559* and *Bra040764* had the highest expression in the flower and callus, respectively. Thirty-six *BrGRP* genes all were expressed in six tissues, whereas 7 *BrGRP* genes were specifically expressed in one or few tissues (Figure 3B). In detail, *Bra008020* was only expressed in flower, *Bra038836* in flower and silique, and *Bra028693* in callus and root. *BrGRP* genes had various expression levels in different tissues, suggesting that they played various roles in organ development and other relevant biological processes.

In addition, *BrGRP* genes were also differentially expressed in two development stages. Ten *BrGRP* genes from classes I, II, IVa, or IVc, were found in the rosette and folding leaves; nine *BrGRP* genes (*Bra035944*, *Bra031784, Bra022895*, *Bra020903*, *Bra014000*, *Bra013176*, *Bra011655*, and *Bra008205*) showed higher expression levels in rosette leaves than in folding leaves, whereas *Bra030284* showed higher expression only in folding leaves (Figure 3C and Supplementary Table S6). Three *BrGRP* genes (*Bra03594*4, *Bra014000* and *Bra030284*) from Subclass IVa – showed differential expression patterns in rosette and folding leaves. Therefore, the above results suggested that *BrGRP* genes might play different roles during the two development stages.

#### Temperature Stress

**FIGURE 4:**
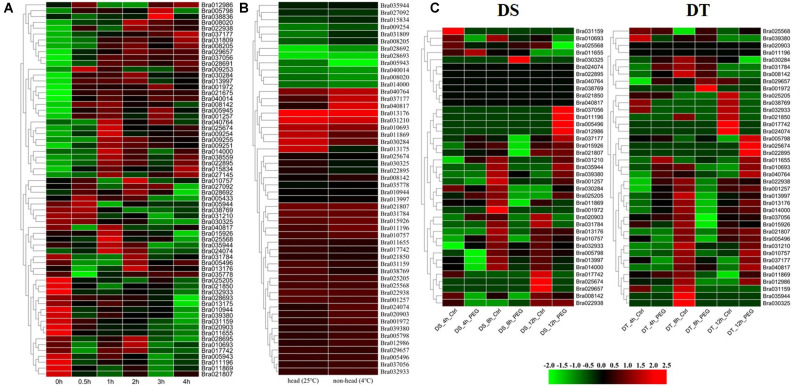
Expression analysis of *BrGRP* genes in Chinese cabbage. **(A)** Differential expression pattern of *BrGRP* gene in Chinese cabbage under 45°C heat stress for 0.5, 1, 2, 3, and 4 h. **(B)** Differential expression pattern of the *BrGRP* gene in Chinese cabbage under low-temperature stress at 25 and 4°C. **(C)** Differential expression profiles of *BrGRP* genes in drought-sensitive (DS) and drought-tolerant (DT) cultivars after drought treatment 4, 8, and 12 h. Ctrl represents the control group without treatment; PEG represents the treatment group under drought stress.

The expression levels of 64 *BrGRP* genes were compared in “Chiifu” seedlings at 0, 0.5, 1, 2, 3, and 4 h after 45°C, respectively (Dong et al., 2015; Figure 4A and Supplementary Table S7), and were divided into 31 expression patterns by STEM software (Supplementary Table S8). Among them, seven expression patterns (profiles 24, 39, 43, 44, 46, 47, and 49) with a total of 20 *BrGRP* genes were up-regulated at five time points under heat stress. Eight *BrGRP* genes in profile 49 were significantly up-regulated five times under heat stress. Fourteen *BrGRP* genes with four expression patterns (profiles 0, 1, 8, and 10) were down-regulated five times under heat stress. Another 20 expression patterns showed disordered fluctuating. For example, *Bra035944* of profile 15 was down-regulated at 0.5, 3, and 4 h, and up-regulated at 1 and 2 h of heat stress. *Bra025568* of profile 30 was down-regulated at 0.5, 2, and 4 h, and up-regulated at 1 and 3 h. *Bra027092* of profile 34 was down-regulated at 1 and 4 h, and up-regulated at 0.5, 2, and 3 h.

A total of 52 *BrGRP* genes were found to express in two true leaves based on the transcriptome data of 29-day-old leaves under 4°C (Zhang et al., 2016). Among them, the expression of 26 genes was significantly different with the low-temperature treatment than with the control treatment (25°C) [log2fold-change (FC)>1]: 17 genes were up-regulated and the remaining nine were down-regulated (Figure 4B and Supplementary Table S9).

#### Drought Stress

The root transcriptome data of Chinese cabbage (CR2355 and ATC92037) were also analyzed to study the expression pattern of *BrGRP* genes under drought stress (Guo et al., 2017). CR2355 is a drought-tolerant (DT) material that can keep the required biomass for mature plants when suffering from transient drought stress during the reproductive phase, while ATC92037 is a drought-sensitive (DS) material which showed a significant reduction in biomass after transient drought stress. After simulating drought stress with 2.5% PEG 6000 in seedlings, 39 *BrGRP* genes were found to differentially express at 4, 8, and 12 h between the DT and the DS materials, and then divided into 11 and 14 profiles, respectively (Figure 4C and Supplementary Tables S10,S11). Among them, for little difference in the expression levels among *BrGRP* genes, there were 6 genes (*Bra040764*, *Bra038769*, *Bra021850*, *Bra040817*, *Bra024074*, and *Bra022895*) *–* in ATC92037 (DS), while *Bra020903* and *Bra011196* in CR2355 (DT). Certainly, some genes showed the same expression pattern between DS and DT, e.g., nine *BrGRP* genes (*Bra001257*, *Bra021807*, *Bra010757*, *Bra031210*, *Bra015926*, *Bra013176, Bra035944*, *Bra011809*, and *Bra031784*) shared profiles 1, 4, 5, and 6. Profile 4 represented continuous down-regulation at 4, 8, and 12 h and profiles 1, 5, and 6 showed continuous down-regulation during the first 8 h of drought stress, after which it was up-regulated until 12 h. Five *BrGRP* genes (*Bra001972*, *Bra025205*, *Bra022938*, *Bra005798*, and *Bra030325*) showed different expressions between DT and DS materials. *Bra005798* was down-regulated or its expression level was not significantly changed in DS material, but it was up-regulated in DT material after drought treatment. *Bra011196* was up-regulated in DS plants but down-regulated or did not change in DT plants after drought treatment. The different expression patterns of these *BrGRP* genes in two materials under drought stress may play different roles in the response of drought tolerance in Chinese cabbage.

#### Soft Rot

**FIGURE 5:**
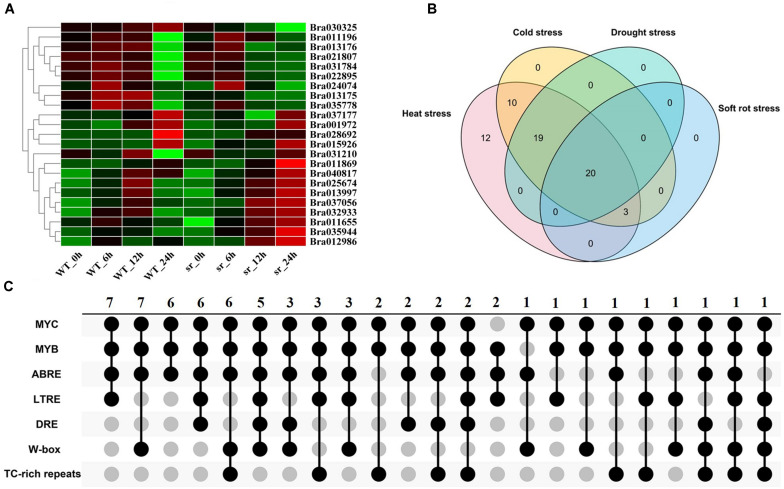
Expression analysis of *BrGRPs* under soft rot stress **(A)**. The numbers of *BrGRPs* involved in various stresses showed by a Venn diagram **(B)**. The numbers of the stress-related *cis-*acting elements in the promoter regions of *BrGRPs* showed by the Venn diagram **(C)**. MYC (CANNTG), MYB (C/TAACNA/G), ABRE (ABA-responsive element, ACGT), LTR (low-temperature-responsive element, CCG AAA), DRE (CCGAC), W box (TTGACC), TC rich repeats (GTTTTCTTAC). Numbers represent the number of involved genes.

To determine the expression of *BrGRP* genes response to soft rot, the transcriptome data from soft rot-resistant mutant (*sr*) and wild control (inbred line “A03”)were used at 0, 6, 12, and 24 h after inoculation with soft rot, respectively (Liu et al., 2019). As Figure 5A and Supplementary Table S12 shown, a total of 23 *BrGRP* genes were identified, which were divided into 12 expression patterns (Supplementary Table S13). Most of *BrGRP* genes were down-regulated within 12 h after inoculation, and up-regulated at 12–24 h (profiles 14, 18, 21, and 25). Four *BrGRP* genes (*Bra011196*, *Bra013997*, *Bra035944*, and *Bra011655*) were up-regulated four times after inoculation in *sr* mutant. These results suggested that *BrGRP* genes had a special response to pathogens.

In summary, Venn diagram revealed the number of *BrGRP* genes involved in various stress responses (Figure 5B and Supplementary Table S14). Twenty *BrGRP* genes (e.g., *Bra025674*, *Bra011869*, and *Bra015926*) showed differential expressions under four types of stresses. Ten *BrGRP* genes (e.g., *Bra028693* and *Bra010944*) responded to heat and cold stresses at different expression levels. Only three *BrGRP* genes (*Bra035778*, *Bra028692*, and *Bra013175*) were induced simultaneously by r heat, cold and soft rot stresses. Furthermore, 12 *BrGRP* genes were only differentially expressed under heat stress. Meanwhile, we found that the expression patterns of 64 *BrGRP* genes were not completely consistent under different abiotic stresses. For example, *Bra030284* was up-regulated under both high- and low-temperature stresses, but showed up-regulated expression in drought-stressed DS plants and down-regulation in DT plants. *Bra010693* was up-regulated under low temperature and drought in DT plants, but down-regulated under high temperature and drought in DS plants. *Bra011869* was down-regulated in low temperature, high temperature, and drought in DS material, but up-regulated under drought in DT material and soft rot. The differential expression of these *BrGRP* genes under various stresses suggested their different functional dissimilation, but needs further validation.

### Analysis of *Cis*-Acting Elements in the Promoter Region of *BrGRPs*

Most *BrGRP* genes were identified under various stresses, so the stress-related *cis-*acting elements were further analyzed. From Figure 5C and Supplementary Table S15), many stress-related *cis-*acting elements were found in the promoter regions of *BrGRP* genes, including DRE, MYB, MYC, ABRE, LTRE, W-box, and TC-rich repeats. DRE has been identified as a *cis-*acting element involved in drought, high salt, and low-temperature stresses, and MYB involved in drought, cold, and salt stresses (Dai et al., 2007). MYC is involved in drought and ABA (Onishi et al., 2006) and LTRE in low-temperature stress (Maestrini et al., 2009). W-box can be combined by WRKY transcription factors to participate in plant responses to stresses such as diseases, drought, and ABA (Wang et al., 2009). TC-rich repeats participate in plant defense and stress response (Banerjee et al., 2013).

The number of stress-related *cis-*acting elements in the promoter region of the 64 *BrGRP* genes was varied from four (*Bra032933*) to 34 (*Bra009254*) and the promoter of each *BrGRP* gene distributed 3–6 types of *cis-*acting elements. The promoter region of 98.4% (63/64) *BrGRP* genes contained MYC element and MYB element. ABRE element was regarded to involve in ABA and drought stress (Manavella et al., 2008), which was located on 89.1% (57/64) of *BrGRP* gene promoters. LTRE element accounted for 50% (32/64) *BrGRP* gene promoters, moreover, DRE, W-box, and TC-rich repeats for 31.3% (20/64), 46.9% (30/64), and 34.4% (22/64) of *BrGRP* gene promoters, respectively. The *BrGRP* genes whose promoters contained the *cis-*acting elements played an important role in the response to adversity stresses.

The promoter regions of each *BrGRP* gene contained several types of stress-related *cis-*acting elements. For example, *Bra031159*, *Bra037056*, and five other genes only had ABRE, LTRE, MYB, and MYC. *Bra028691*, *Bra028692*, and five other genes only had ABRE, MYB, MYC, and W-box. Differences in *cis-*acting element types s on the *BrGRP* gene promoter regions may be associated with different expression patterns among *BrGRP* genes under biotic and abiotic stresses. However, the number of stress response *cis-*acting elements in the promoter region is not completely consistent with the expression patterns of *BrGRP* genes under stress. For example, ABRE element is related to drought (Manavella et al., 2008) but *Bra028691* containing 11 ABRE elements were induced by high-temperature stress. Consequently, to elaborated the gene function need to further explore the regulation mechanism of transcription factors.

### Two *BrGRP* Genes Participated in NaCl Stress

**FIGURE 6:**
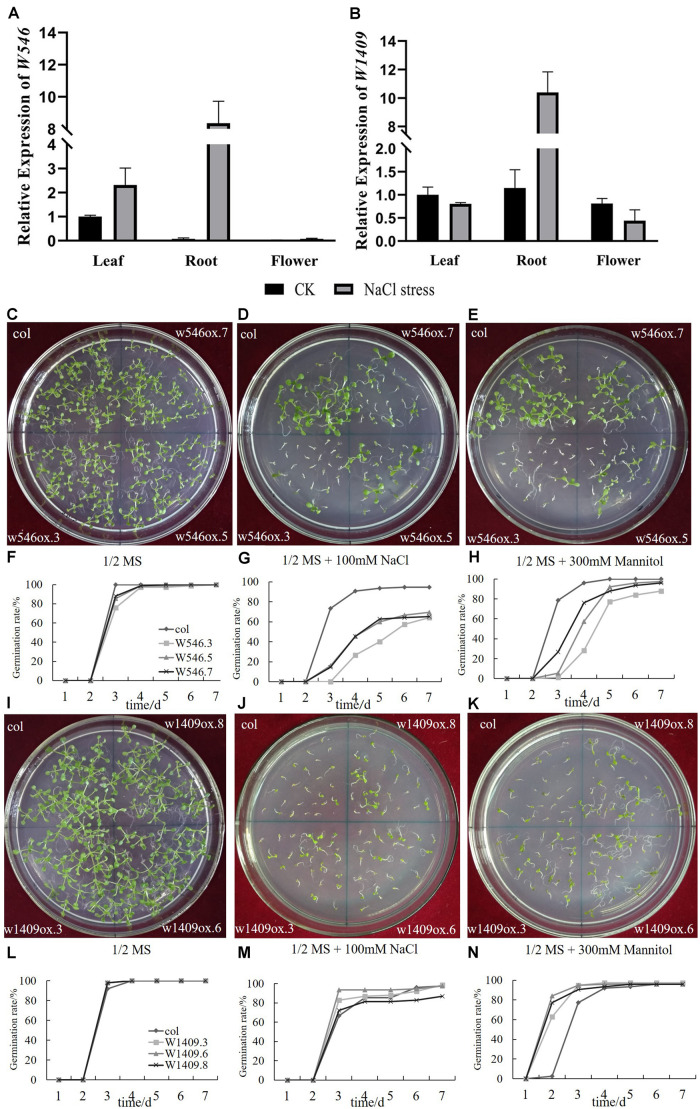
Two *BrGRP* genes involved in NaCl stress. **(A,B)** Expression analysis of two *BrGRP* genes *w546* (*Bra030284*) and *w1409* (*Bra014000*) in three tissues, leaf root and flower, of Chinese cabbage under salt stress by qRT-PCR analysis. Data were normalized with the *GAPDH* gene and vertical bars indicate standard deviation. **(C–E)** the photo of the seed germination of *w546* -overexpressed *Arabidopsis* under control, NaCl and mannitol treatments. **(F–H)** The statistic results of **(C–E)**. **(I–K)** the photo of the seed germination of *w1409* -overexpressed *Arabidopsis* under control, NaCl and mannitol treatments. **(L–N)** The statistic results of **(I–K)**. WT: wild type. w546ox.3, w546ox.5, w546ox.7 are three individual transgenic lines with the *w546* overexpressed; w1409ox.3, w1409ox.6, and w1409ox.8 are three individual transgenic lines with *w1409* overexpressed.

In our study, *w546* (*Bra030284*) was mainly expressed in the leaves, but clearly lower expression in the roots and flowers, accounting for 3–7% of the leaves (Figure 6A). After 7 days under salt stress, the expression of *w546* was all up-regulated in three tissues (up to 114 times in roots) of Chinese cabbage.

Although the expression of *w1409* was the highest among 64 BrGRP genes in leaves, but without clear expression differences in the three tissues (Figure 6B). Under salt stress, the expression of *w1409* was highly up-regulated in the roots (nine times higher than in the control roots), but was slightly repressed in the leaves and flowers. The results revealed that the two *BrGRP* genes were induced under salt stress. Although, *w546* and *w1409* were both from Subclass IVa, their expression levels were still different, suggesting that they respond differently to abiotic stress.

To further clarify the role of the two *BrGRP* genes in responses to NaCl and mannitol stress, seed germination was measured in three independent lines of transgenic *Arabidopsis* plants. The results showed that there was no clear different in germination rate among three transgenic *w546* lines (w546ox.3, w546ox.5, and w546ox.7) and the control wild-type (WT) in a normal environment or without any stress, although the seed germination potential was a little lower than that of the control WT (Figures 6C,F). However, 100 mmol⋅L^–1^ NaCl significantly inhibited the seed germination of w546ox transgenic lines with, less than 66%, significantly lower than that of the WT (100%); the germination potential was less than 16%, far lower than that of the WT (73.33%) (Figures 6D,G). For 300 mmol⋅L^−1^ mannitol treatment, the final germination rate of three transgenic *w546* lines was not much different from that of the control, but the seed germination potentials of the w546ox lines were much less than 1/3 of the WT (which was about 78.67%); even the germination potentials of w546ox.5 and w546ox.3 were 0 (Figures 6E,H). Therefore, an overexpressed *w546* gene could significantly inhibit seed germination in transgenic *Arabidopsis* under salt stress, and the different phenomena between NaCl and mannitol stress suggested that Na^+^ damage may occur by osmotic stress.

Like with *w546*, the seed germination rate of w1409ox lines were also not significantly different from that of the WT without stress (Figures 6I,L). Under the 100 mmol⋅L^–1^ NaCl treatment, the germination potentials of w1409ox.8 were not significantly different from that of the control, but the germination potential of w1409ox.3 and w1409ox.6 were 82.67% and 93.33%, respectively, which were higher than WT (66.67%). However, there was no significant difference in germination rate between three transgenic lines and the WT in the end, except for w1409ox.3 (Figures 6J,M). Under the 300 mmol⋅L^−1^ mannitol treatment, the germination potential of w1409ox.3 and w1409ox.6 was both 94.67%, and that of W1409ox.8 reached 90.67%, which were all higher than that of the WT (77.33%), although the final germination rate was not significantly different (Figures 6K,N), which was similar to the result under NaCl stress. Therefore, overexpressed *w1409* may significantly accelerate seed germination rate in transgenic lines under NaCl and mannitol stress treatments.

In summary, overexpression of two *BrGRP* genes had the opposite effects on seed germination under 100 mmol⋅L^–1^ NaCl and 300 mmol⋅L^−1^ mannitol stresses, although *w546* and *w1409* both belonged to the same subfamily of Subclass IVa *RBP-GRP*, which suggested different functions between w546 and *w1409*.

## Discussion

The plant *GRP* gene family is a superfamily with a glycine repeat (Gly) n-X domain. However, due to the diversification of their protein domains, gene expression patterns, and subcellular localization, these *GRP* genes are sometimes not considered a gene superfamily, but a group of proteins with some repeating structural motifs (Kar et al., 2012). This may change the classification of the *GRP* gene family. Fifteen, 22, 12, and 18 glycine-rich RNA-binding proteins (RBGs) have been identified in *Arabidopsis*, Chinese cabbage, rice and maize genomes, respectively (Zhang et al., 2014; Krishnamurthy et al., 2015). Furthermore, 9 and 51 *GRP* genes have been identified in sweet potato and *Curcuma longa L*., respectively (Kar et al., 2012; Lu et al., 2019). In our study, 64 *BrGRP* genes were preliminarily identified in Chinese cabbage based on BRAD genome sequence, and were divided into five classes according to their conserved domains, which was similar to the classifications in previous studies (Fusaro et al., 2001; Bocca et al., 2005).

The Chinese cabbage genome not only underwent three genome-wide replication events, which also occurred in other cruciferous plants (Thomas et al., 2006; Franzke et al., 2011) but can also be divided into three subgenomic groups, LF, MF1, and MF2, according to the number of genes lost (Wang et al., 2011; Cheng et al., 2013). In this study, the syntenic analysis of *GRP* genes between Chinese cabbage and *Arabidopsis thaliana* also verified this genome-wide replication event, confirming that Chinese cabbage originated from a hexaploid ancestor, and underwent rearrangement to become diploid after chromosome fusion (Wang et al., 2011). Meanwhile, 64 *BrGRP* genes were found in Chinese cabbage, including 20 LF genes, 23 MF1 genes, and 18 MF2 genes, which were not in a three-fold relationship with the 41 *GRP* genes of *Arabidopsis*. This may suggest that *BrGRP* genes in Chinese cabbage still evolved after the genome-wide replication event, and a larger-scale loss-of-function event occurred, preventing functional redundancy.

*GRP* gene expression in *Arabidopsis thaliana* is tissue- or organ-specific. *GRP* genes in classes I and II are mainly expressed in seeds, siliques, roots, and leaves; class III has the highest expression in shoot tips, rosettes, seeds, and flowers; class IV is highly expressed in seeds, siliques, rosettes, and flowers; and Subclass V is up-regulated only in inflorescences (Vivek et al., 2015; Czolpinska and Rurek, 2018). This was not completely consistent with the expression of *BrGRP* genes in various tissues of Chinese cabbage, class I was mainly expressed in roots and leaves; class II was mainly expressed in root, stem, leaves and flowers; class IV had higher expression levels in various tissues; and classes III and V had lower or no expression in all tissues. *GRP* gene classes I and II may act as the active components of plant cell walls and play a crucial role in plant cell growth and organ differentiation (Park et al., 2001). *BrGRP* gene class IV has strong RNA recognition and binding abilities, and may participate in the molecular process of plant growth and development by activating splicing or regulating transcription (Czolpinska and Rurek, 2018). In the study, we found that the *BrGRP* genes that were differentially expressed at various developmental stages of Chinese cabbage belonged to classes I, II, and IV, which is similar to previous studies (Winter et al., 2007; Vivek et al., 2015) and might act similar functions in terms of growth and development.

Plant *GRP* genes that can be induced by various stresses may play another role in plant resistance. Among 8 glycine-rich RNA-binding protein genes (*AtGR-RBP1–AtGR-RBP8*) reported in *Arabidopsis thaliana*, all except for *AtGR-RBP5* and *AtGR-RBP6* were strongly expressed by low-temperature stress (Kim et al., 2005; Kwak et al., 2005). Under drought and salt stresses, *AtGR-RBP1* expression increased, but the expression of *AtGR-RBP4* and *AtGR-RBP7* gradually decreased, whereas that of *AtGR-RBP5* and *AtGR-RBP6* did not change (Kwak et al., 2005). In tobacco, the expression of *NtGRP1* was induced and continuously increased during the first 24 h of waterlogging stress, and then decreased; it was present at low levels under high or low temperature, drought, high salt, and ABA stress (Lee et al., 2009; Chen et al., 2010). *NtRGP2* and *NtRGP3* were also expressed by waterlogging and high and low-temperature stresses, but were not affected by ABA treatment with 100 μmol⋅L^−1^ (Chen et al., 2010). Among the four glycine-rich RNA-binding protein genes (*OsGR-RBP1-OsGR-RBP4*) in rice, only *OsGR-RBP4* was expressed by high temperature, high salt, and drought stresses (Sahi et al., 2007). In this study, a total of 64 *BrGRP* genes were identified as being differentially expressed under high temperature, low temperature and drought stresses, and soft rot. Although the *BrGRP* genes expressed in biotic and abiotic stresses were different, and a total of 64, 52, 39, and 23 of *BrGRP* genes were induced under high temperature, low temperature, drought, and soft rot treatment, respectively.

The same *BrGRP* gene also showed different expression dynamics under different stresses, suggested that they had multiple expression patterns and different functions under adversity stress in Chinese cabbage. *AtRZ-1* was strongly expressed by low temperature and freezing stress, but was negatively regulated during seed germination and seedling growth under drought and high salt stress (Kim et al., 2005; Kim Y.O. et al., 2007; Kim and Kang, 2006). However, overexpression of *AtRZ-1B* and *AtRZ-1C* or loss of function mutations do not affect *Arabidopsis* seed germination or seedling growth under these same stress conditions (Kim et al., 2010). *AtRZ-1B/1C* regulate RNA splicing, gene expression, and many key aspects of plant development via interactions with proteins, such as SR (Wu et al., 2016). Here, most *BrGRPs* were identified to take part in stress responses, and a large number of stress-related elements were identified in their promoter region, suggesting an inextricable link and different response mechanisms between *BrGRP* genes and abiotic stress. Furthermore, two genes in Subclass Iva, *w546* (*Bra028063*) and *w1409* (*Bra014000*), were significantly up-regulated under salt stress, and their expression levels were significantly different during seed germination in transgenic *Arabidopsis* plants under salt and mannitol stresses, suggesting that *BrGRP* genes had different molecular responses to various stresses. It is very interesting that there was no clear growth or developmental defect in the two types of transgenic plants, suggesting that they can tolerate stress well.

## Conclusion

In this study, 64 *BrGRP* genes were identified in the Chinese cabbage genome based on the 41 *AtGRP* genes in *Arabidopsis*. The *BrGRP* genes in Chinese cabbage were mainly composed of alkaline hydrophilic stable proteins and are secreted outside the cell membrane and nucleus, with only a few found in organelles such as mitochondria and chloroplast. *BrGRP* genes were divided into five classes, and shared close relationships with their homologs in *Arabidopsis*. Chromosomal localization of these *BrGRP* genes and syntenic analysis with *Arabidopsis thaliana* strongly confirmed that Chinese cabbage did undergo a genome-wide triple duplication event during its evolution. The specific expression of these genes was evaluated under various stresses, and 3–6 types of response stress *cis-*acting elements in the promoter region of these *BrGRP* genes were also identified, suggesting that they have potential roles in plant stress responses. Based on the effects of *BrGRP* gene expression in various tissues of Chinese cabbage and the germination of *Arabidopsis* strains overexpressing two *BrGRP* genes under abiotic stress, we found that the expression of *BrGRP* genes in Chinese cabbage is induced by abiotic stress. *Arabidopsis* lines overexpressing *BrGRP* genes accelerated or inhibited seed germination under abiotic stress, but which *BrGRP* genes might play more important regulating mechanism remained unclear in response to biotic and abiotic stresses.

## Data Availability Statement

The datasets presented in this study can be found in online repositories. The names of the repository/repositories and accession number(s) can be found in the article/Supplementary Material.

## Author Contributions

XL and XX conceived and designed the experiments. XL and YC wrote the manuscript. XL, YC, and MG were responsible for data analysis. ML provided helpful advice on data analysis. ML and XX revised the manuscript and supervised the research. All authors read and approved the final manuscript.

## Conflict of Interest

The authors declare that the research was conducted in the absence of any commercial or financial relationships that could be construed as a potential conflict of interest.

## Footnotes

1 http://brassicadb.org/brad/

2 http://www.arabidopsis.org/

3 http://www.ncbi.nlm.nih.gov/cdd

4 http://pfam.xfam.org/

5 http://web.expasy.org/protparam/

6 http://linux1.softberry.com/

7 http://rice.plantbiology.msu.edu/

8 https://maizegdb.org/

9 https://www.cottongen.org/

10 http://sweetpotato-garden.kazusa.or.jp/

11 http://meme-suite.org/tools/meme

12 http://bioinformatics.psb.ugent.be/webtools/plantcare/html/
