# Supplementary material for: Molecular Characterization, Expression Pattern and Function Analysis of Glycine-Rich Protein Genes Under Stresses in Chinese Cabbage (Brassica rapa L. ssp. pekinensis)
Source: Front Genet. 2020 Jul 23;11:774. doi: 10.3389/fgene.2020.00774 (PMC7396569; doi:10.3389/fgene.2020.00774)
Supplement: Supplementary file 2 [file Presentation_1.pdf]

Supplementary Material

1 Supplementary Data

Supplementary Material should be uploaded separately on submission. Please include any supplementary data, figures and/or tables. All supplementary files are deposited to FigShare for permanent storage and receive a DOI.

Supplementary material is not typeset so please ensure that all information is clearly presented, the appropriate caption is included in the file and not in the manuscript, and that the style conforms to the rest of the article. To avoid discrepancies between the published article and the supplementary material, please do not add the title, author list, affiliations or correspondence in the supplementary files.

2 Supplementary Figures and Tables

2.1 Supplementary Figures

Figure S1 LOGO of GRP conservative motifs in Chinese cabbage.

Figure S2 Phylogenetic analysis of GRP proteins in Chinese cabbage (red gene ID) Arabidopsis (black gene ID), rice (yellow gene ID), maize(purple gene ID), *Gossypium raimondii* (blue gene ID), *Gossypium arboretum* (green gene ID), and sweet potato(orange gene ID). (A) Phylogenetic analysis of GRP proteins constructed with the Maximum likelihood method. (B) Phylogenetic analysis of GRP proteins constructed with the Neighborhood-joining method.

2.2 Supplementary Tables

Table S1 Primer sequences used in this study.

Table S2 Information on *BrGRP* genes in Chinese cabbage.

Table S3 Distribution of *BrGRP* genes in the genome of Chinese cabbage.

Table S4 Orthologous pairs of *GRP* genes between Chinese cabbage and *Arabidopsis thaliana*.

Table S5 FPKM values of *BrGRP* genes in five different tissues of Chinese cabbage.

Table S6 Expression values of *BrGRP* genes at different development stages of Chinese cabbage.

Table S7 Expression values of *BrGRP* genes under 45°C heat treatment in Chinese cabbage.

Table S8 Expression profiles of *BrGRP* genes under 45°C heat treatment in Chinese cabbage.

Table S9 Expression values of *BrGRP* genes under low temperature treatment in Chinese cabbage.

Table S10 Expression values of *BrGRP* genes under drought treatment in Chinese cabbage.

Table S11 Expression profiles of *BrGRP* genes under drought treatment in Chinese cabbage.

Table S12 Expression values of *BrGRP* genes under soft rot treatment in Chinese cabbage.

Table S13 Expression profiles of *BrGRP* genes under soft rot treatment in Chinese cabbage.

Table S14 Same *BrGRP* genes expressed under different biotic and abiotic stresses

Table S15 Quantitative statistics of cis-acting elements related to adversity stress in the promoter region of *BrGRP* genes
