# Supplementary figures and images for: Molecular Characterization, Expression Pattern and Function Analysis of Glycine-Rich Protein Genes Under Stresses in Chinese Cabbage (Brassica rapa L. ssp. pekinensis)
Source: Front Genet. 2020 Jul 23;11:774. doi: 10.3389/fgene.2020.00774 (PMC7396569; doi:10.3389/fgene.2020.00774)

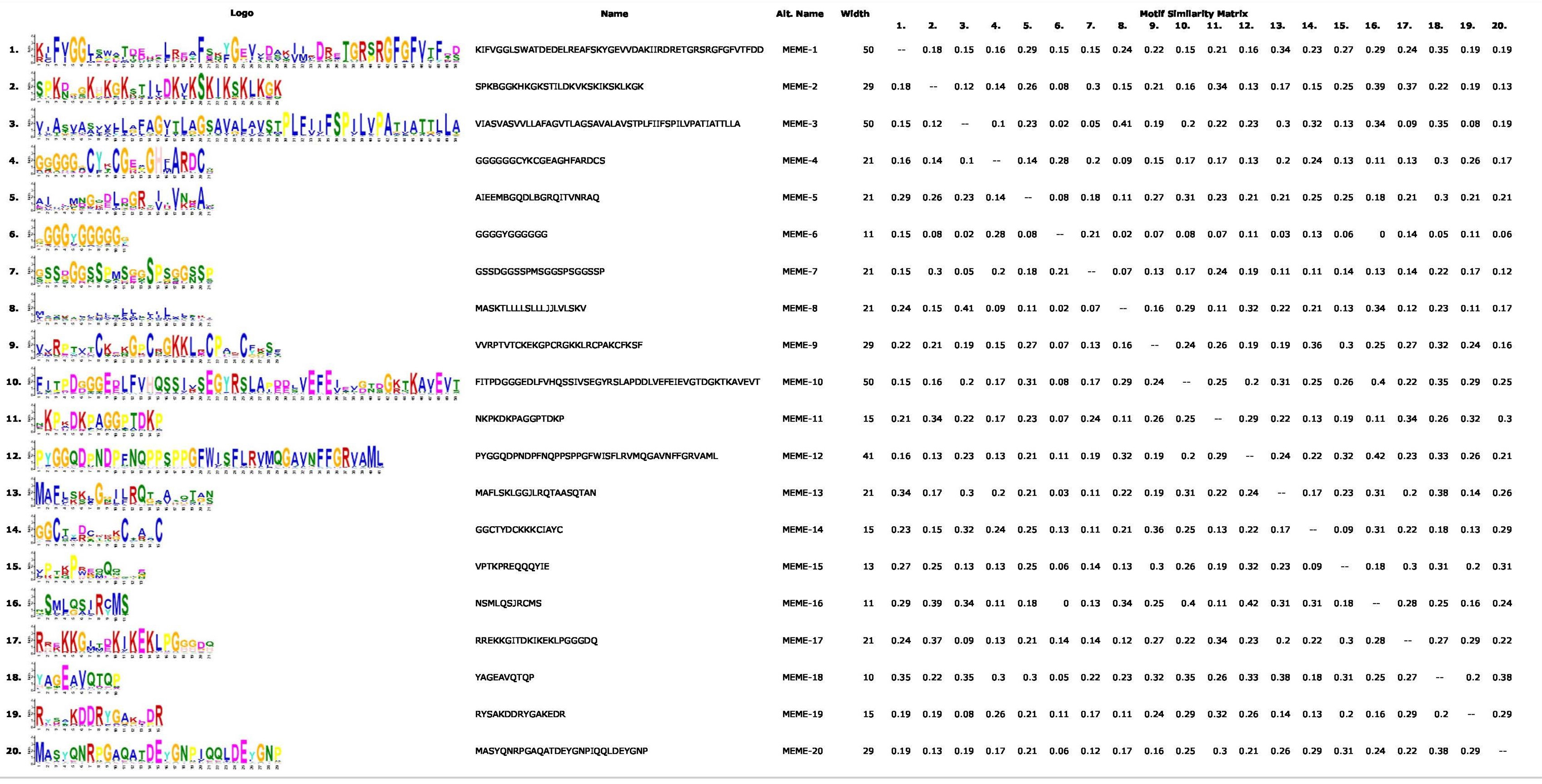

Supplement: Supplementary file 3 [file Image_1.jpg]

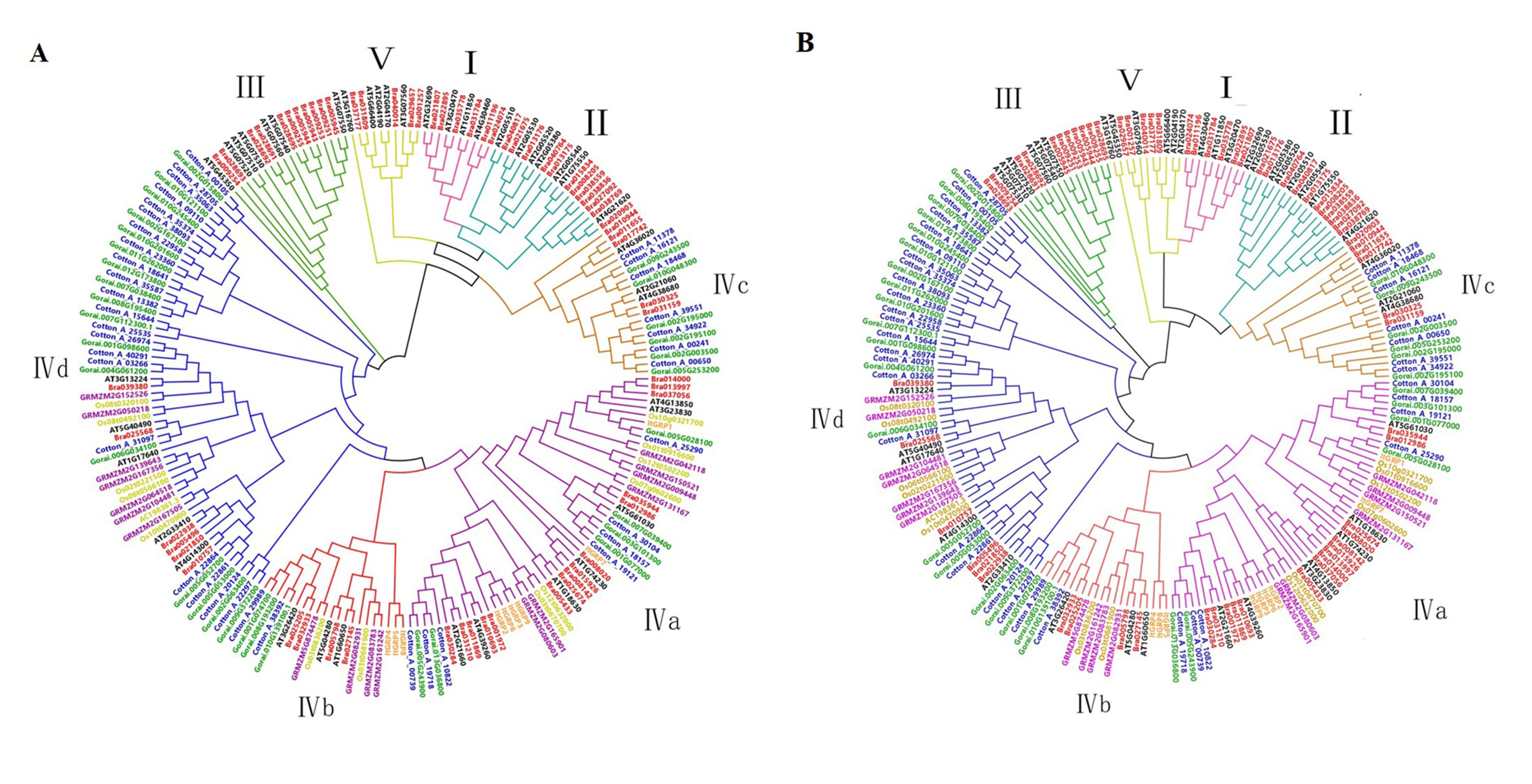

Supplement: Supplementary file 4 [file Image_2.jpg]
